# Support Networks and Family Empowerment in Early Intervention

**DOI:** 10.3390/ijerph19042001

**Published:** 2022-02-11

**Authors:** Gabriel Martínez-Rico, Cecilia Simón, Margarita Cañadas, Robin Mcwilliam

**Affiliations:** 1 Campus Capacitas, Catholic University of Valencia, 46001 Valencia, Spain; margarita.canadas@ucv.es; 2 Interfaculty Department of Developmental and Educational Psychology, Autonomous University of Madrid, 28049 Madrid, Spain; 3 Department of Special Education and Multiple Abilities, University of Alabama, Tuscaloosa, AL 35487, USA; ramcwilliam@ua.edu

**Keywords:** early childhood, family outcomes, family supports, empowerment, family-centered practices

## Abstract

Despite the importance of empowerment and the support network of families who receive early intervention (EI) with a family-centered approach, there is little evidence of a relationship between these two variables and family characteristics that might influence this relationship. This study analyzes the correlations between the perception of empowerment of the families, the family supports used, and the socio-demographic factors of both the child and the family. The study consisted of 44 families who received family-centered EI services. Our results show that families mainly used formal supports, followed by informal supports, and, to a lesser extent, intermediate supports. This indicates that families with children who receive EI preferably use the support network based on EI programs, schools, and professionals. Along with this formal support network, primary caregivers rely on their partners, parents, or friends—that is, the informal support network. Family empowerment was not correlated with age, diagnosis, or the reason for referral to EI; on the other hand, it was related to the supports where the families with the lowest empowerment scores were those who made greater use of formal support over informal support. Early intervention professionals must know, from the first encounter, the type and level of support of each family to enhance the development of the child and promote empowerment in families.

## 1. Support Networks and Family Empowerment in Early Intervention

The use of family-centered practices in early intervention (EI) contribute to positive outcomes for both the family and the child [1,2]. Families’ achievements are reflected in the extent of their empowerment, which had an impact on their competencies and confidence in the care and development of their child, their ability to identify their rights, and their capacity to use their support networks and community services [3,4,5].

One of the key factors in intervention regarding the empowerment of families is the use of formal and informal support networks [6,7]. The presence of these support networks is associated with family well-being [8]. In fact, the quality of support networks and the received emotional support are predictors of the stress level in parents of children with disabilities [9]. 

Despite these studies, the literature shows the need to examine factors related to family empowerment, particularly the role of formal and informal support networks, with the aim of promoting effective intervention strategies [10]. In this manuscript, support networks (formal, intermediate, and informal) are considered the set of supports that families use and that they perceive as such.

In the last decade, many EI services have implemented family-centered practices [11,12]. As shown in this work, family-centered practices orient part of their objectives to empower families and increase their network of supports. Therefore, in addition to studies of the development of children, measurement of the quality of life of the family and their empowerment as outcomes of the intervention has become of interest. Specifically, this study is focused on the empowerment of families who receive family-centered services and the relationship between this empowerment and family support networks. Next, we will try to conceptualize the two central constructs of the study: family empowerment and social support. 

### Family Empowerment and Support Networks

From an ecological perspective of EI, the level of empowerment and the size and strength of family support networks are indicators of the success of the intervention, that is, important outcomes of services [13]. What do we mean by family empowerment? The conceptualization of family empowerment in this study is based on the proposal made in this regard by Fernandez-Valero, Serrano, Cañadas, and McWilliam [4], who defined family empowerment as families’ perception of their skills, self-confidence, and knowledge about the care and development of their child with special needs, to achieve satisfying family functioning. This empowerment will be reflected in the achievement of certain outcomes by families.

Therefore, according to Bailey and Bruder [14], family outcomes are the benefits that families receive after using a service, such as receiving information about the child’s disability, being able to explain their child to people in their support networks, being able to advocate for quality services, and effectively promoting their child’s development. 

In this context, it is understood that social supports have an impact on families, regardless of whether they have children with disabilities or not [15,16].

Social networks have a direct or indirect influence on the functioning of both the family and the child [17,18]. For example, as pointed out by Espe-Sherwindt and Serrano [18] from the results of different research with families who have children with disabilities, high levels of social support correspond to low levels of parental stress, and this is especially critical in early intervention [18,19]. Indeed, social support is considered to be one of the most powerful external resources for coping with stress in parents who have children with disabilities [9,18].

What do we mean by “social supports”? Based on previous proposals, such as the one made by Dunst, Trivette, and Cross [20], Woodman [9] defines social support as “a multidimensional construct that includes physical and instrumental assistance, resource sharing, and emotional and psychological support” (p. 41).

The family support networks are considered to be the set of social supports that families use and that they are perceived as such. These networks can be divided into three types of supports: informal, intermediate, and formal. The informal supports include, e.g., grandparents, parents-in-law, other extended family, neighbors, colleagues, and friends, while the intermediate support are made up of associations, church members, etc., and finally, formal supports consist of EI professionals, pediatricians, teachers, specialized physicians, other members of the community, etc. [7,15]. In a study conducted in the USA by Edwards [21], over 80% of the participating families identified, as their main supports, informal caregivers, such as grandparents and friends, followed by intermediate supports, such as groups of parents of other children, and last, formal supports provided by professionals. Moreover, in the study conducted by Más, Giné, and McWilliam [13], Spanish families identified the extended family, particularly grandparents, as the main source of emotional support. 

Not all types of support, however, are perceived to be equally useful by families or have the same relevance to family empowerment. Families with more solid support networks are more effective at facing new challenges [22]. According to some studies, informal supports influence family outcomes to a greater extent than formal supports do [6,15]. In turn, intermediate supports (i.e., non-professional supports provided by the community) are related to greater family competence [16]. 

It is also important to differentiate between the amount and usefulness of these supports. A large support network is not necessarily more useful than a smaller one. Only the family can determine the quality of its support networks and how useful they are [23]. 

In this sense, the nature of their interactions with formal supports—that is, professionals (EI, schools, health centers, education, etc.) relates to the level of family empowerment. In fact, the type of relationship that is established between professionals and families is a mediating factor between the supports provided by the services and the perception of family quality of life (FQoL) [24,25]. When these interactions reflect a collaborative relationship [26], they have a positive impact on FQoL [24]. Hence, we have also paid attention to this aspect in this research.

Nevertheless, although research has shown the importance of supports and their relationship to empowerment, the literature shows cultural differences when it comes to delimiting which supports are important to families and, thus, are related to FQoL or families’ needs (e.g., Australia, Singapore, Japan, or Spain) [27,28,29,30,31,32].

In this sense, it is necessary to expand the field of research on the empowerment of families receiving EI and its relationship with support networks [3,33]. The study of these two variables is very relevant, both for their theoretical importance and their impact on the improvement of services.

Based on the need to investigate this issue in different cultural contexts, the objectives of the present study were the following:

(1) Analyze the characteristics of empowerment perceived by families in the EI and the factors that influence its development.
(2) Analyze the types of formal, intermediate, and informal supports used by families and their frequency and level of perceived support.
(3) Study the relationship between family empowerment and different types of support used.

## 2. Method

### 2.1. Participants

A total of 44 families of children who receive EI participated in the study. These families were selected according to the following inclusion criteria: (a) the child was receiving family-centered EI service based on a family-centered approach, (b) they voluntarily agreed to participate, and (c) all families were attending EI services for the second year in a row. 

The participants were the primary caregivers of the children. Table 1 shows the characteristics of both the caregivers and the children they took care of. Forty-four families with children from 2 to 6 years of age who receive AT have participated, and six families did not meet the criteria to participate in the study. The mean age of the children is 4.02 and the standard deviation is DS = 1.191. The families attended early intervention services integrated into the Recommended Practices program of the Plena Inclusion confederation. This program is continuously evaluated to measure the extent to which the practices focus on the family [11]. 

The special educational needs (SEN) diagnosis was established, in all cases, by the official, specialized teams in charge of this evaluation (early intervention teams). Once the managers of the EI service agreed to participate in the study, families were invited to participate. Families completed the paper questionnaires and returned them in a closed envelope to the researchers. The questionnaires included information about the study, the reassurance about voluntary participation, and the anonymity and confidentiality of the information gathered. The study was conducted with the approval of the Ethics Committee of the Autonomous University of Madrid. 

### 2.2. Measures

The instruments used to gather the information were the following:

*Family Outcomes Survey* (FOS) [34]. The FOS was validated in Spain by Fernández-Valero, Serrano, Cañadas, and McWilliam [3]. This scale considers “outcomes” as the families’ perception of their competence, self-confidence, and knowledge about the care of their child with disabilities to achieve satisfactory family functioning [34]. It consists of 20 items, in a Likert scale: 1 = totally disagree to 5 = totally agree. Section A of the FOS, which has been validated with Spanish families, captures the construct of empowerment based on five outcomes determined from the impact of EI services on the following family outcomes: (a) understanding the strengths, needs, and abilities of the child; (b) knowing their rights and advocating for the child; (c) helping the child to develop and learn; (d) creating a support system; (e) having access to the community. 

A reliability analysis (Cronbach’s alpha) was performed for each subscale: understanding the strengths (α = 0.631), knowing their rights (α = 0.805), helping the child to develop (α = 0.788), creating support systems (α = 0.463), and having access to the community (α = 0.695). The results showed that the internal consistency was high for the subscales “knowing their rights” and “helping the child to develop”, moderate for “understanding the strengths” and “gaining access to the community”, and low for “creating support systems”. The definition of family empowerment is based on the work of Fernandez-Valero, Serrano, Cañadas, and McWilliams [3]. These authors define empowerment as the perception that families have about their skills, degree of self-confidence, and knowledge about the care and development of their child with special needs, in order to achieve satisfactory family functioning.

Therefore, the last subscale was considered only in descriptive analyses. This study began with the definition of family empowerment proposed by Fernandez- Valero, Serrano, Cañadas, and McWilliam [3], who defined family empowerment as families’ perception of their skills, self-confidence, and knowledge about the care and development of their child with special needs to achieve satisfying family functioning.

*Family Support Scale* (FSS) [35]. This scale measures the usefulness of different sources of support available to the primary caregiver of the child with disabilities in the 3–6 months before the completion of the survey. This tool is an adaptation of the version that was validated with Spanish families [3]. The scale consists of 19 items, organized as a Likert scale based on the extent of support perceived (1 = no help, 5 = extremely useful). The scale includes an additional option to state that the support was not available. The three dimensions of the FSS are informal supports (extended family or family friends), intermediate supports (neighbors, colleagues, and family-related social groups), and formal supports (educational, social, health resources). A reliability analysis was conducted (Cronbach’s alpha) for each subscale, showing good internal consistency for all three dimensions: informal supports (α = 0.826), intermediate supports (α = 0.801), and formal supports (α = 0.745). 

*Socio-demographic data protocol.* This protocol gathered descriptive information about the families (age, existence of children with disabilities in the family, education level of the mother and father, etc.). It also gathered information about the child with disabilities (age, gender, associated disorders, percentage of disability, received supports, etc.).

SPSS was used (v25.0, SPSS Inc., Chicago, IL, USA) for statistical analysis. For the descriptive results, absolute and relative frequencies (percentages) were determined for the categorical variables, and means and standard deviations were calculated for the quantitative variables. Spearman’s rho correlations were used to analyze the relationship between empowerment and support networks. The Mann–Whitney U-test was used to analyze the differences between means of empowerment, supports, and demographic variables of the families and children who receive EI. Cohen’s *δ* (Cohen, 1988) was also computed for these analyses, considering *δ* ≥ 0.2, *δ* ≥ 0.5, and *δ* ≥ 0.8 as low, medium, and large effect sizes, respectively (Cohen, 1992).

## 3. Results

### 3.1. Families Outcomes

The results obtained in the scale show a mean response score close to the maximum score, which indicates a high perception of empowerment by the families: understanding strengths (M = 18.83); knowing their rights (M = 16.58; helping the child (M = 18.55), support system (M = 17.73) (see Table 2). The differences between understanding strengths and the other three dimensions were all noteworthy but of different effect sizes: knowing their rights: large (d = 1.16); helping the child: small (d = 0.23); support system: moderate (d = 0.63). The differences between knowing their rights and the other two dimensions were similarly noteworthy: helping the child: large (d = 0.93) and support system: small (d = 0.49). Finally, the difference between helping the child and support system was small (d = 0.43). Therefore, the FOS dimensions were sensitive to different aspects of family empowerment.

Table 3 shows the mean, standard deviation, and number of respondents for all items.

### 3.2. Supports Used by the Families 

The study analyzed the support network that families counted on, distinguishing between formal, informal, and intermediate supports (see Table 4). 

The FSS captures families’ ratings of the usefulness of supports and the frequency of use. Table 4 shows that the most useful supports are formal supports, followed by informal supports. In this case, the frequency of use of intermediate supports was substantially lower. 

Families with children receiving EI services aged between birth and 6 years rated EI programs, the school, and both EI professionals and teachers as the most frequently used. This formal support network is also useful with other health professionals, such as physicians and social workers, although at a lower frequency and use (Table 5). 

Nevertheless, although research has shown the importance of supports and their relationship to empowerment, the literature shows cultural differences when it comes to delineating which supports are important to families and, thus, are related to FQoL or families’ needs (e.g., Australia, Singapore, Japan, or Spain) [26,27,28,29,30,31].

As can be observed, the main support network in early childhood is based on EI. Together with this network of formal supports, the main caregivers rely on their spouse or partner, their parents, or their friends—that is, the extended family that makes up the informal support network. The importance of these supports is determined, in part, by close and trusting relationships between the primary caregiver and the spouse/partner, grandparents, or friends.

The least used and valued support according to the families is the parents and friends of the partner. 

Last, it is worth pointing out that the family support network had few intermediate supports. The most used supports, although to a lesser extent than formal and informal supports, were social groups, other, colleagues, and parent groups. Neighbors and the church were not rated highly useful. 

### 3.3. Factors Associated with Family Empowerment: Supports

This section examines correlations between the five dimensions of the family empowerment scale, measured through the Family Outcome Survey (FOS), and the three dimensions of the support network, measured through the FSS.

The division between high and low empowerment has been made on the basis of the median value following the procedure established by DeCoster, Gallucci, and Iselin, [36].

The correlation analysis (Table 6) shows that lower use of intermediate supports by families is associated with higher scores on dimensions 1 (understanding the strengths) and 3 (helping the child to develop). It should be noted that this correlation does not occur with the FOS scale as a whole.

### 3.4. Supports Used as a Function of Socio-Demographic Variables

In this study, none of the analyzed socio-demographic variables showed statistically significant differences in the empowerment and support networks of the families that use EI services. Specifically, the study analyzed the influence of the child’s age, diagnosis, education level of the main caregiver, age of the caregivers, and having another child with disabilities. 

The Mann–Whitney U-test was performed to evaluate the possible relationships between types of family support and the empowerment subscale “helping the child to develop” (Table 7 and Table 8). The results show that the families with low scores in this subscale of empowerment made greater use of intermediate supports (Z = −2.161; *p* = 0.031; *δ* = 0.53). Although the difference was not statistically significant, it was observed that the families with a lower score in the empowerment subscale “helping the child to develop” tended to make greater use of supports in general (i.e., the total score for the scale), with a medium effect size (Z = −1.726; *p* = 0.084; *δ* = 0.51) (Table 8). 

## 4. Discussion

This study describes the extent of the empowerment perceived by families who use EI services (0–6 years) as well as the characteristics of the frequency and extent of their use of support networks. Moreover, this work examines the relationship between different components of family empowerment and different types of support (formal, informal, and intermediate) in families that use family-centered EI. 

In this investigation, the primary caregivers receiving family-centered EI services perceived their empowerment to be high; that is, they felt they were competent in and self-confident about the care and development of their children with disabilities. Although causality cannot be shown, these results show that in general, families who receive family-centered services in EI experience a positive impact on empowerment. This result is consistent with the other study [27], who found a high level of empowerment in the families receiving family-centered EI services. Similar results have been reported in other studies [30,32]. 

The results of the present study show that families who attend early intervention services for the second consecutive year perceive their competence and confidence in caring for their children as high, especially in “understanding the strengths, needs, and abilities of the child.” Families felt capable of meeting the needs of their children. They did not feel as competent, however, in their ability to advocate for their children and family. These results strongly align with Adams [27].

Unsurprisingly, the families show a high level of empowerment in general because they participate in services with family-centered practices, but, what factors are related to less empowerment in these families? Our results showed the child-related variables (age, diagnosis, or reason for referral to early care) were not associated with differences in empowerment, but the supports used by families with lower empowerment scores made greater use of intermediate supports. Similarly, families who rated greater empowerment in the dimension “helping their child” (i.e., those who considered themselves more capable of helping their child to develop, learn new abilities, satisfy his/her needs and learn through routines) found intermediate supports less useful.

Support networks are a fundamental factor for intervention [6,9]. Our results show that families mainly used formal supports, followed by informal supports, and, to a lesser extent, intermediate supports. This indicates that families with children who receive EI preferably use the support network based on EI programs, schools, and professionals. Along with this formal support network, primary caregivers rely on their partners, parents, or friends—that is, the informal support network. The importance of these supports is determined by trust relationships.

These results are partially in line with those found by Edwards [21] in a USA survey, in which over 80% of the families identified informal caregivers (parents and friends) as the main support network, followed by intermediate supports (groups of parents), and last, formal support networks of professionals. The study of Más, Giné, and McWilliam [13], with Spanish families attending services with a family-centered approach, also identified the extended family as the main source of emotional support, especially the grandparents of the child. 

The support networks of the families of children with disabilities are a determining variable in family empowerment, influencing their participation in social and community activities [37,38,39]. This study shows that the families with greater empowerment make less use of informal and intermediate supports.

Family outcomes of empowerment did not show a statistically significant relationship with the age of the child, diagnosis, or the reason for referral to EI. Dempsey and Dunst [40] had similar results. Some studies, however, have reported a significant relationship between empowerment and parent-level variables. Kalleson, Jahnsen, and Østensjø [41] found an association between the education level of the mother and perceived empowerment, with mothers having a higher education level reporting lower empowerment. Wakimizu, Fujioka, Yoneyama, Iejima, and Miyamoto [42], in a study with Japanese families, found lower empowerment levels in families who had more children, in younger families, and in the families with little knowledge of available informal supports. 

It is worth highlighting that the results obtained in this study have a direct impact on the professional practices of EI services, showing the importance of expanding the focus of the intervention to other caregivers who might contribute to improving family outcomes in addition to child outcomes [42,43,44,45]. Professionals should include practices to identify potential family support networks and may ask families to complete the Family Support Scale used in our study at the beginning of the intervention. 

## 5. Limitations

Considering that few services in Spain employ a totally family-centered approach, more services and research into the supports (formal, intermediate, and informal) available to families and their perceived usefulness would be useful.

It is also necessary to broaden the sample and promote studies that analyze in depth the changes that occur in the course of early intervention, both in empowerment and in the support network of families.

Regarding the limitations of the study, the analyses carried out do not allow us to establish causal relationships between the support and empowerment variables. Given the importance of this aspect from both a theoretical and an applied point of view, the directionality of the relationships between family empowerment and perceived supports should be understood more deeply to determine any possible causal effect between the two.

Neither have been considered in the analyses the influence of certain sociodemographic variables of the families and their impact on family empowerment and the use of supports; these are aspects that should be considered in future studies. It is necessary to expand this research to determine the extent to which child characteristics (diagnosis, need for support, level of functionality, age of the child) influence the variables under study. Similarly, caregiver characteristics (sex, age, studies carried out, employment situation) and their influence on the perception of family empowerment and the use of supports should also be studied.

Finally, the results obtained do not allow us to conclude the extent to which family-centered practices affected empowerment compared to non-family-centered practices. This should be another objective of future studies using a larger sample size.

## 6. Conclusions

Early childhood professionals should consider the positive relationship between empowerment and family support networks. A greater network of supports promotes positive outcomes for both the child and the family. 

Family outcomes were generally high in all the dimensions studied, with the lowest scores, however, in those corresponding to knowing their rights and support system. The supports among parents in the same situation through intermediate supports allow them to share their problems, so the creation of parent groups in their community could be promoted. Including the extended family (e.g., grandparents) in those groups is a good option for everyone to know the disability, which would allow them to support the parents and actively participate in the care of the children [45,46]. It is also important to help families identify intermediate or formal supports to reduce the possible stress, even if they do not consider these as generally useful supports.

Professionals should understand the benefits of family support and promote empowerment in their practices. Future studies could further examine empowerment and the usefulness of supports, including other factors related to the child, such as the intensity of the required supports or the characteristics of the environment, such as rurality (i.e., rural and urban areas).

## Figures and Tables

**Table 1:** Child and caregiver characteristics.

Demographic Variables	N (%)
Respondent	
Mother	32 (72.72)
Father	12 (27.27)
Age of the respondent	
30–39 years	5 (11.3)
40–49 years	31 (70.4)
50–59 years	8 (18.1)
Number of siblings	
0	21 (48.8)
1	16 (37.2)
2	4 (9.3)
3	2 (4.7)
Number of members of the family unit	
2	1 (2.3)
3	21 (48.8)
4	15 (34.9)
5	6 (14.0)
Primary caregiver	
Mother	1 (2.3)
Father	2 (4.5)
Both	41 (93.2)
Another child with disabilities	
No	41 (93.2)
Yes	3 (6.8)
Another family member with a disability	
No	35 (79.5)
Yes	8 (18.2)
Age of the child who receives EI	
2	2 (4.5)
3	17 (38.6)
4	10 (22.7)
5	8 (18.2)
6	7 (15.9)
Sex of the young child with a disability	
Female	9 (24.3)
Male	28 (75.7)
Associated difficulties	
Yes	15 (34.1)
No	29 (65.9)

**Table 2:** Perceived family empowerment.

Measures		Understanding Strengths	Knowing Their Rights	Helping the Child	Support System
N	Valid	44	44	44	44
M		18.837	16.585	18.559	17.739
SD		1.349	2.549	1.674	2.157
Minimum		16.00	8.00	14.00	10.00
Maximum		20.00	20.00	20.00	20.00

**Table 3:** Results of empowerment by dimensions and items.

Item	Dimension 1: Understanding the Strengths, Needs, and Abilities of the Child	M	SD	N
1	We know the next steps to follow in the development and learning of our child	4.372	0.716	44
2	We know the strengths and abilities of our child	4.791	0.406	44
3	We know the difficulties or needs of our child	4.698	0.507	44
4	We can see the progress of our child	4.977	0.157	44
	Dimension 2: Knowing their rights and defending the interests of their child			
5	We can find and use services and programs that are available to our child	4.318	0.638	44
6	We know our rights regarding the special needs of our child	4.068	0.789	44
7	We know who we should contact and what to do when we have doubts or concerns	4.628	0.682	44
8	We know the available options for our child once he/she finishes the program	3.571	1.037	44
	Dimension 3: Helping the child to develop and learn			
9	We can help our child to get along well with others	4.535	0.623	44
10	We can help our child to learn new abilities	4.756	0.419	44
11	We can help our child to satisfy his/her needs	4.634	0.517	44
12	We can work on the objectives of our child during the daily routines	4.634	0.560	44
	Dimension 4: Creating support systems			
13	We feel comfortable when speaking with our relatives and friends about the needs of our child	4.205	0.904	44
14	We have friends or relatives who listen to and care about us	4.791	0.592	44
15	We can talk to other families who have children with similar needs	4.233	1.053	44
16	We have friends or relatives whom we can count on when we need help	4.512	0.872	44
	Dimension 5: Accessing the community			
17	Our medical and dental needs are satisfied	4.651	0.642	44
18	The needs related to the care of our child are satisfied	4.698	0.551	44
19	Our transportation needs are satisfied	4.698	0.665	44
20	Our feeding, clothing and housing needs are satisfied	4.791	0.592	44

**Table 4:** Perceived usefulness of the support network.

Factors	N	Mean	SD	Minimum	Maximum
Formal support	41	3.871	0.807	2	5.00
Informal support	41	3.325	0.898	2	5.00
Intermediate support	36	2.255	1.171	1	5.00
Total support	36	9.486	2.001	6.28	13.75

**Table 5:** Informal, intermediate, and formal support networks.

Support Networks	Items	% Unavailable	M	SD	N
Formal supports	Physician	4.5%	2.971	1.328	41
	EI program	0%	4.463	1.027	41
	School	0%	4.109	0.943	41
	Professionals	4.5%	4.393	0.958	41
	Professional public health agencies	11.4%	3.391	1.142	41
Informal supports	My parents	13.6%	4.343	0.968	41
	My parents-in-law	27.3%	2.966	1.614	41
	Relatives/siblings	22.7%	3.110	1.316	41
	Relative/siblings of my partner	22.7%	2.482	1.481	41
	Partner	2.3%	4.475	0.905	41
	Friends	6.8%	3.049	1.001	41
	My partner’s friends	27.3%	2.573	1.371	41
	My children	40.9%	2.432	1.304	41
Intermediate supports	Neighbors	27.3%	1.768	1.247	41
	Other parents	27.3%	2.371	1.402	41
	Colleagues	27.3%	2.287	1.284	41
	Groups of parents	40.9%	2.210	1.307	41
	Social groups	47.7%	2.375	1.510	41
	Church	59.1%	1.834	1.273	41

**Table 6:** Relationships between empowerment and the support network.

FOS Dimensions	Formal Supports	Informal Supports	Intermediate Supports	Total Supports
1. Understanding the strengths	−0.033	−0.118	−0.327 *	−0.194
2. Knowing their rights	0.051	−0.013	−0.076	0.011
3. Helping the child to develop	−0.086	−0.186	−0.329 *	−0.280
5. Accessing the community	0.204	0.222	−0.082	0.121

* *p* < 0.05.

**Table 7:** Relationship between high and low empowerment and the support network.

Support Networks	FOS 3 Helping the Child to Develop	N	Mean Rank	Sum of Ranks
FSS Informal supports	Low	22	22.66	498.50
	High	19	19.08	362.50
	Total	41		
FSS Intermediate supports	Low	22	24.75	544.50
	Hight	19	16.66	316.50
	Total	41		
FSS Formal supports	Low	22	21.73	478.00
	High	19	20.16	383.00
	Total	41		
FSS Total supports	Low	22	24.00	528.00
	High	19	17.53	333.00
	Total	41		

**Table 8:** Support network: informal supports, intermediate supports, and formal supports.

	FSS Informal Supports	FSS Intermediate Supports	FSS Formal Supports	FSS Total Supports
Mann–Whitney U	172.500	126.500	193.000	143.000
Wilcoxon’s W	362.500	316.500	383.000	333.000
Z	−0.955	−2.161	−0.420	−1.726
*p*	0.340	<0.05	0.674	0.084

## Data Availability

You can find a further description of the data in Analysis and evaluation of the process of inclusive education for students with Autism Spectrum Disorders from early childhood education to university: social participation as the hub of analysis (EITEA). https://www.equidei.es/ (accessed on 17 December 2021).
